# 3D Point Cloud Recognition Based on a Multi-View Convolutional Neural Network

**DOI:** 10.3390/s18113681

**Published:** 2018-10-29

**Authors:** Le Zhang, Jian Sun, Qiang Zheng

**Affiliations:** 1 State Key Laboratory for Strength and Vibration of Mechanical Structures, School of Aerospace, Xi’an Jiaotong University, Xi’an 710049, China; lezhang0829@stu.xjtu.edu.cn (L.Z.); yongzhoucaomin@stu.xjtu.edu.cn (Q.Z.); 2 Shaanxi Engineering Laboratory for Vibration Control of Aerospace Structures, Xi’an Jiaotong University, Xi’an 710049, China

**Keywords:** multi-view, point clouds, convolutional neural network, recognition

## Abstract

The recognition of three-dimensional (3D) lidar (light detection and ranging) point clouds remains a significant issue in point cloud processing. Traditional point cloud recognition employs the 3D point clouds from the whole object. Nevertheless, the lidar data is a collection of two-and-a-half-dimensional (2.5D) point clouds (each 2.5D point cloud comes from a single view) obtained by scanning the object within a certain field angle by lidar. To deal with this problem, we initially propose a novel representation which expresses 3D point clouds using 2.5D point clouds from multiple views and then we generate multi-view 2.5D point cloud data based on the Point Cloud Library (PCL). Subsequently, we design an effective recognition model based on a multi-view convolutional neural network. The model directly acts on the raw 2.5D point clouds from all views and learns to get a global feature descriptor by fusing the features from all views by the view fusion network. It has been proved that our approach can achieve an excellent recognition performance without any requirement for three-dimensional reconstruction and the preprocessing of point clouds. In conclusion, this paper can effectively solve the recognition problem of lidar point clouds and provide vital practical value.

## 1. Introduction

Currently, as an efficient and rapid remote sensing technique, the lidar system has been widely applied to object recognition, detection, and tracking. The advent of lidar has altered the way that humans obtain information. People used to obtain information from two-dimensional images dominated by optics. At present, they can get it from three-dimensional point clouds with spatial coordinates. Because lidar has the advantages of high ranging accuracy, large measuring range, strong directionality and not being affected by environmental conditions, it has wide applications in the fields of unmanned driving [1,2], robotics [3,4], and aerospace [5,6]. However, point cloud processing has become a challenging research task due to large data volume, irregular data format, and high scene complexity. In recent years, deep learning [7] has gradually emerged and has made major breakthroughs in related fields such as image classification [8,9,10], speech recognition [11,12,13], and text processing [14,15]. Deep learning, through an end-to-end model, directly performs a nonlinear transformation layer by layer on the original input from a lower level to a higher level. Hence, it can automatically extract the information contained in massive data, which greatly simplifies the learning process. With the arrival of the big data era and the development of hardware devices such as the Graphics Processing Unit (GPU), deep learning will have stronger expressive capabilities and wider applications. Briefly, the use of deep learning to recognize lidar point clouds has become one of the most important research topics.

Compared with two-dimensional images, three-dimensional point clouds can provide the spatial geometric information of objects, which will not be affected by conditions such as light. However, there are certain difficulties in using deep learning to process 3D point clouds.

- The point cloud is not in a regular format. Unlike an image, a point cloud is a set of points distributed in space. Hence, it is non-grid data. However, typical convolutional architectures can merely deal with highly regular data formats, and it is, therefore, hard to use convolutional neural networks to process raw point clouds.
- The point cloud is unordered. There are various orderings of points, indicating that there are many matrix representations of a particular point cloud. Therefore, how to adapt the changes of the waypoints are arranged is a problem in point cloud processing.
- Three-dimensional point clouds normally contain only the spatial coordinates of points, lacking rich textures, colors, and other information.

In addition, lidar can simply scan within a certain field angle and obtain more abundant information by moving to different field angles in practical applications. Therefore, it is of great practical significance to recognize multi-view point clouds. This paper proposes a novel representation of 3D point clouds with 2.5D point clouds from multiple views. Then, it designs a point cloud recognition model based on a multi-view convolutional neural network, which can directly process the raw 2.5D point clouds of all views jointly. Our model consists of three key parts: the feature extraction structure to extract the features of point clouds, the view fusion network to merge features of 2.5D point clouds from all views into a global feature descriptor, and a classifier composed of fully connected layers to perform classifications. The feature extraction structure gathers the transformation correction network, the convolution layers, and the symmetric module in the integrale whole. Next, we describe its structure. First, two transformation correction networks generate affine transformation matrices acting on the input point clouds and exacted features; this makes them able to take a good deal of the situation of an affine transformation. Then, two convolution layers are used to capture the deeper features. In the end, the symmetric module based on the max pooling layer is used to aggregate the information from all points. We observe a significant improvement brought about by the symmetric module, the transformation correction network, and the view fusion network through experiments. Furthermore, we also conduct extensive experiments to study the influence of the number of points and views, which guide us how to choose an appropriate point cloud resolution and the number of views according to technical index requirements in practical applications.

**Figure 1:**
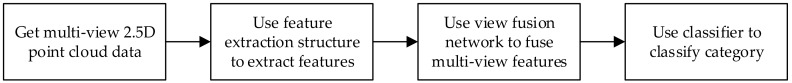
The system flowchart.

The system flowchart is shown in Figure 1.

This paper is structured as follows. Section 2 reviews the related work on 3D data recognition using deep learning. Section 3 describes the form of multi-view 2.5D point cloud data and its generation method. Section 4 proposes a recognition model based on multi-view convolutional neural network and makes a detailed introduction. Section 5 examines the proposed method using multi-view 2.5D point cloud data corresponding to ModelNet10 and ModelNet40. Section 6 presents the conclusions and outlook.

## 2. Related Work

At present, there are a large variety of shape representations for using deep learning to recognize 3D data. Next, we discuss various shape descriptors and their limitations.

Some existing methods focus on transforming three-dimensional point clouds or objects into voxel grids, which can be deemed as the promotion of two-dimensional pixels in three-dimensional space. Danil Prokhorov [16] represented the original data as the density of points on the 3D voxel grids occupied by the object. Daniel Maturana et al. [17] proposed the volumetric occupancy grid which represented the estimate of spatial occupancy as its value, including three different occupancy grid models: the binary occupancy grid, the density grid, and the hit grid. Zhirong Wu et al. [18] suggested representing 3D shapes as a probability distribution of binary variables on the 3D voxel grids. Each grid was represented as a binary tensor which is given a value depended on whether the voxel grid is inside or outside the shape. A volumetric representation can completely preserve shape information of three-dimensional data. However, the higher resolution introduces an increase in calculation and processing time, while the lower resolution brings about quantization errors and limits the discrimination of learned features. Besides that, points mostly distribute on the surface of the three-dimensional shape, which makes numerous voxels be empty.

Another method is to take the raw coordinates of 3D point clouds as input so as to classify the point clouds through an integrated network architecture. Charles R.Qi et al. [19] took the coordinates of point clouds as the input and designed PointNet based on the convolutional neural network in accordance to the characteristics of 3D point clouds, making the network able to deal with irregular point cloud data. However, PointNet can merely handle a 3D point cloud from the whole object without taking into account the fusion of multiple views.

A few others have represented 3D point clouds or shapes with 2D images. Hang Su et al. [20] obtained 12 images by setting 12 virtual cameras every 30 degrees to render the object. Konstantinos sfikas et al. [21] represented the 3D object as three images of 2D panoramic views corresponding to the *X*, *Y*, and *Z* axes. Guan Pang et al. [22,23] projected a 3D point cloud into 2D images based on the depth at different viewpoints. Xiaozhi Chen et al. [24] proposed a more compact representation by projecting a 3D point cloud to the bird’s eye view and the front view which are encoded by height, intensity, and density. Although this type of shape descriptor significantly reduces the computation complexity, it inevitably loses part of the shape information and requires additional pre-processing, which does not ensure the instantaneity of recognition.

## 3. Point Cloud Data

### 3.1. Point Cloud Data Representation

A point cloud is a set of three-dimensional points and its mathematical expression is shown as follows:

*M =* {*P_i_*(*x*, *y*, *z*), *i* = 0, 1, …, *N* − 1} (1)

where *M* denotes the point set, *P* represents a point which is represented by its coordinates (*x*, *y*, *z*), *i* represents the serial number of some point, and *N* is the total number of points in some point cloud.

### 3.2. Multi-View 2.5D Point Cloud Data Generation

**Figure 2:**
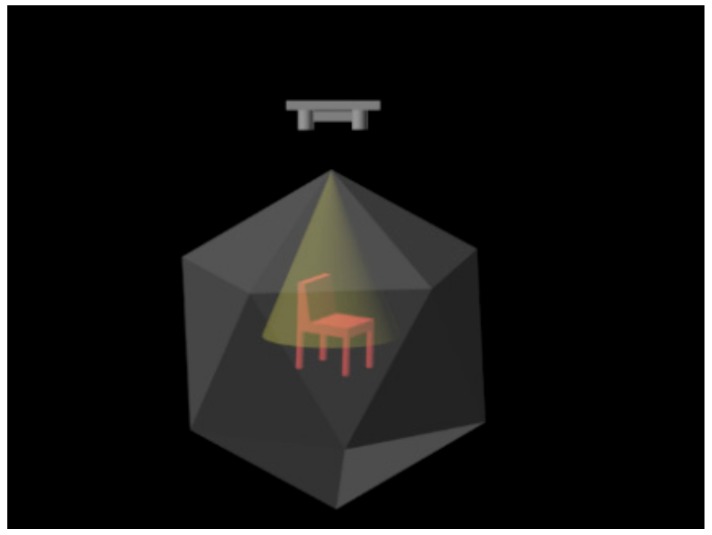
The generation schematic diagram of multi-view 2.5D point cloud data.

**Figure 3:**
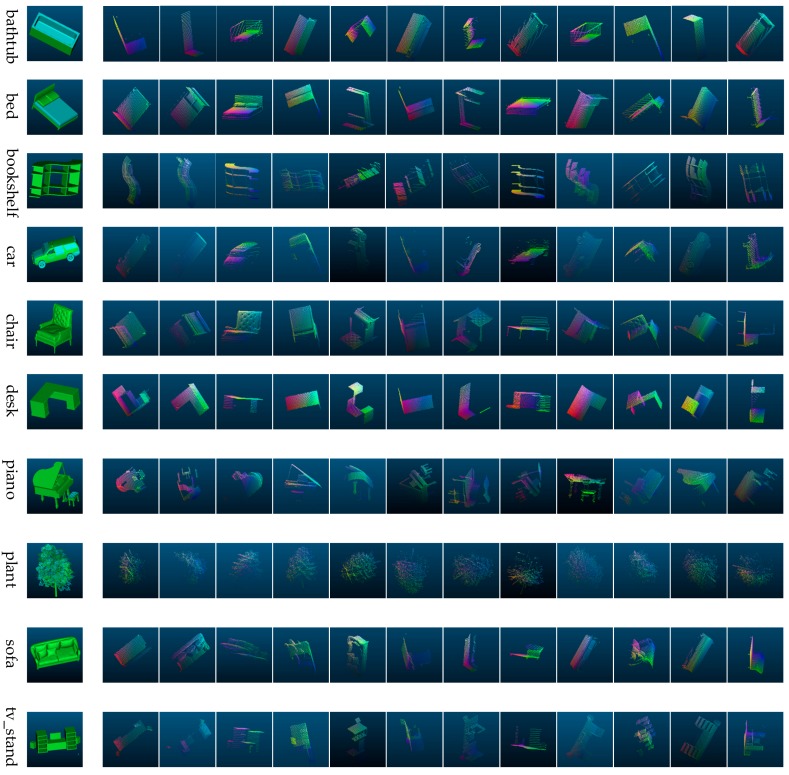
The CAD (Computer Aided Design) models and their multi-view 2.5D point cloud data. We set the value of the R (red), G (green), and B (blue) components corresponding to the coordinate value of the *x*-axis, *y*-axis, and *z*-axis, respectively, in the three-dimensional space, thereby obtaining the color of the point. R, G, and B represent red, green, and blue in the RGB color model in which red, green, and blue light are added together in various ways to reproduce a broad array of colors.

In this paper, we obtain 2.5D point clouds of the Computer Aided Design (CAD) models under different field angles based on PCL. We first place the CAD model in a tessellated sphere building from an icosahedron where the center of the model coincides with the center of the tessellated sphere and then generate multiple 2.5D point clouds by setting the viewpoint locations at the vertices or faces of the tessellated sphere. The selected parameters are listed as follows, the radius of the tessellated sphere is 1 unit, the number of subdivisions applied to the triangles of the original icosahedron is 0, and the field of view of the virtual camera is 45 degrees. By this method, we can get 2.5D point cloud data from 12 views and 20 views when setting the viewpoint locations at the vertices and faces, respectively. The point cloud data generated by this method is named the multi-view 2.5D point cloud data. Figure 2 shows a generation schematic diagram of multi-view 2.5D point cloud data. Figure 3 illustrates part of CAD models we experiment on as well as their multi-view 2.5D point cloud data.

### 3.3. Multi-View 2.5D Point Cloud Data Characteristics

Multi-view 2.5D point cloud data have the following four characteristics:

- Multi-view 2.5D point cloud data are unordered [19]. Multi-view 2.5D point cloud data are a set of points with no specific order.
- There exists a correlation among the points [19]. Each point does not exist in isolation but shares spatial information with nearby points.
- Multi-view 2.5D point cloud data are sensitive to some transformations such as translation, scaling, and rotation. The coordinates of the points change after transformations, but the recognition results should remain unchanged since the shapes of point clouds are invariant.
- Multi-view 2.5D point cloud data are composed of 2.5D point clouds from multiple views. Consequently, each view contains local features that make up global features.

## 4. Methods

### 4.1. Symmetry Module

In order to solve the disorder of some input such as point clouds, Oriol Vinyals et al. [25] proposed the Read-Process-and-Write architecture for unordered input data. Ravanbakhsh et al. [26] proposed deep permutation-invariant networks with a simple permutation equivariant layer to perform the classification on point clouds or other sets where the output is invariant to permutations of input. Charles R. Qi et al. [19] proposed to approximate a general function which can integrate the information of points, and they gave a theoretical analysis and detailed formula deduction. The mathematical expression is as follows:

*f* ({*x*_1_, …, *x_n_*}) ≈ *g*(*h*(*x*_1_), …, *h*(*x_n_*)) (2)

where *x*_1_, …, *x_n_* are input vectors, *h* is a continuous function which is approximated by several convolution layers, *g* is a symmetric function which is approximated by the single variable function and the max pooling layer. Thus we obtain the *f* function named symmetry module.

In fact, the symmetry module is composed of three convolution layers and a max pooling layer, it first captures the features by convolution layers and then achieves the invariance of the output to the input based on the max pooling layer. Similar to the add or multiply operation, no matter how the order of the points changes, we can get the only result after performing the max pooling operation across each feature map obtained by the convolution on point clouds, where the kernel size of max pooling is equal to the size of feature map which it acts on.

### 4.2. The Transformation Correction Network

In order to ensure that the recognition results are invariant to some transformations of input, M Jaderberg et al. [27] used spatial transformation neural networks to correct image transformation through learning the parameters of affine transformations and interpolation. Charles R. Qi et al. [19] proposed using input transformation and feature transformation to rectify the transformations of both input point clouds and the extracted features, respectively. The input transformation takes point clouds as the input and generates an input transformation matrix to perform an affine transformation on input point clouds. The feature transformation takes the extracted features as the input and generates a feature transformation matrix to perform an affine transformation on the extracted features.

An affine transformation is geometrically defined as an affine mapping between two vector spaces, consisting of a non-singular linear transformation followed by a translation transformation. The affine transformation has the general characteristics of converting parallel lines into parallel lines and mapping finite points into finite points, including translation, rotation, and scaling.

The affine transformation in three-dimensional space is shown as

(3) $$\left[\begin{array}{c} x^{\prime }\\ y^{\prime }\\ z^{\prime } \end{array}\right]=\left[\begin{array}{ccc} s_{x} & 0 & 0\\ 0 & s_{y} & 0\\ 0 & 0 & s_{z} \end{array}\right]\left[\begin{array}{ccc} 1 & 0 & 0\\ 0 & \cos\alpha & \sin\alpha \\ 0 & -\sin\alpha & \cos\alpha \end{array}\right]\left[\begin{array}{ccc} \cos\beta & 0 & -\sin\beta \\ 0 & 1 & 0\\ \sin\beta & 0 & \cos\beta \end{array}\right]\left[\begin{array}{ccc} \cos\theta & \sin\theta & 0\\ -\sin\theta & \cos\theta & 0\\ 0 & 0 & 1 \end{array}\right]\left[\begin{array}{c} x\\ y\\ z \end{array}\right]+\left[\begin{array}{c} t_{x}\\ t_{y}\\ t_{z} \end{array}\right]\;$$

where *s_x_*, *s_y_*, *s_z_* are scaling factors of the *x*-axis, *y*-axis, and *z*-axis, accordingly; *α*, *β*, *θ* are the rotation angles along the *x*-axis, *y*-axis, and *z*-axis, respectively; *t_x_, t_y_, t_z_* are the translation factors of the *x*-axis, *y*-axis, and *z*-axis, correspondently. Equation (3) can be written as follows:

**X**’ = **SR*_x_***(***α***)**R*_y_***(***β***)**R*_z_***(***θ***)**X** + **A**
 = **SMX** + **A**
  = **TX**+**A** (4)

Among them, **R***_x_*(*α*), **R***_x_*(*β*), **R***_x_*(*θ*) are the rotation matrices rotating along the *x*-axis, *y*-axis, and *z*-axis, respectively. **X** is the coordinate matrix before the affine transformation, **X**’ is the coordinate matrix after the affine transformation, **S** is the scaling matrix, **M** is the rotation matrix, **A** is the translation matrix, and **T** is the transformation matrix.

Extended by a basic image processing algorithm, named affine transformation, this paper considers designing the transformation correction network to correct affine transformations of point clouds such as translation, scaling, and rotation. The transformation correction network is similar to a micro convolutional neural network and it learns the transformation matrix and the translation matrix adaptively through the back-propagation algorithm.

**Figure 4:**
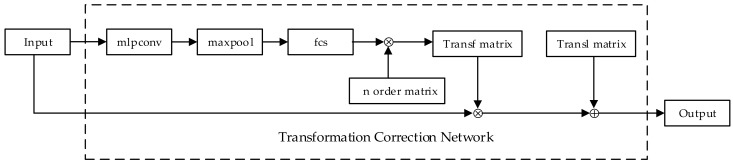
The transformation correction network architecture. In Figure 4, mlpconv is a composition of three convolution layers, maxpool is a max pooling layer, fcs is a composition of two fully connected layers, Transf matrix is the transformation matrix, Transl matrix is the translation matrix, ⊗ represents the matrix multiplication, and ⊕ represents the matrix addition.

The transformation correction network proposed in this paper is shown in Figure 4. It contains three convolution layers, a max pooling layer, and two fully connected layers. Then, it maps the output of the fully connected layers into a regular *n* order transformation matrix. This paper achieves the correction of transformation by multiplying the input by the transformation matrix as well as adding the translation matrix. The transformation correction network can be placed anywhere before the view fusion network in the model. It takes the raw point clouds or the extracted features from each view as input thereby obtaining the transformation matrix and the translation matrix by learning scaling factors, rotation angles, and translation factors. The transformation correction network can adaptively learn the parameters of affine transformation to achieve the correction of various transformed data. Moreover, it can automatically select regions of interest during training, which can enhance the robustness of the model.

### 4.3. The View Fusion Network

The 2.5D point cloud data collected from disparate views contains local features from different regions while the features of a single view lack comprehensiveness. In order to fully express the three-dimensional objects, it is essential to fuse features from all views. Hang Su et al. [20] used a virtual camera to render the CAD model into 12-angle images and utilized the element-wise maximum operation across the views to synthesize the information from all views into a single shape descriptor in the view-pooling layer. This paper designs a view fusion network in front of the classifier, which is similar to a micro neural network [28]. Its formula is as follows:

(5) $$\begin{array}{l} I_{j}^{l}=\sum_{i}w_{ij}v_{i}^{l-1}+\theta_{j}^{l}\\ O_{j}^{l}=\max\left(0,I_{j}^{l}\right) \end{array}$$

where *i* denotes the *i*th neuron of the (*l* – 1)th layer, *j* denotes the *j*th neuron in the *l*th layer, *w_ij_* represents the weight from the *i*th neuron to the *j*th neuron, $\theta_{j}^{l}$ represents the threshold of the *j*th neuron in the *l*th layer, $I_{j}^{l}$ represents the input of the *j*th neuron in the *l*th layer, $O_{j}^{l}$ represents the output of the *j*th neuron in the *l*th layer.

**Figure 5:**
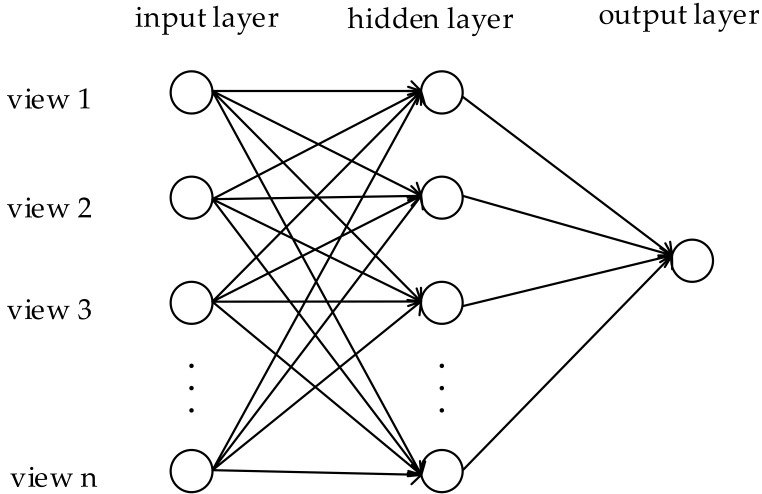
The view fusion network schematic diagram.

As shown in Figure 5, the view fusion network contains three layers, the input layer, the hidden layer, and the output layer. The input matrix is

**V** = [*v*_1_*, v*_2_, …, *v_n_*] (6)

where **V** represents the feature matrix composed of the extracted features from each view, 1, 2, and *n* represent the sequence number of view.

The output layer of the view fusion network has a single neuron. The view fusion network essentially implements a mapping function from the input to output, which can approximate any nonlinear continuous function with arbitrary precision and automatically extract the reasonable rules between input and output. Therefore, it can well map the features from multiple views into a global feature descriptor with a high degree of self-learning and self-adaptive capabilities. Compared with the view-pooling layer proposed by Hang Su et al. [20], the accuracy of view fusion network achieves a remarkable improvement.

### 4.4. The Multi-View Convolutional Neural Network

**Figure 6:**
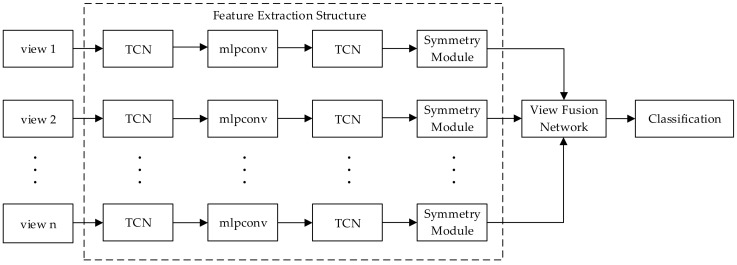
The structural framework of the multi-view convolutional neural network. In Figure 6, TCN represents the transformation correction network and mlpconv represents a composition of two convolution layers.

**Figure 7:**
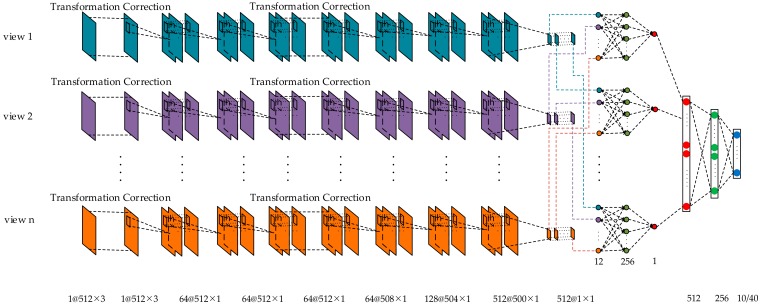
The multi-view convolutional neural network diagram.

As illuminated in Figure 6, the operation to exact features of the multi-view point clouds is simply referred to as the feature extraction structure. The 2.5D point cloud data from each view is fed into the feature extraction structure independently, where all the branches are exactly the same. The feature extraction structure consists of three parts including the transformation correction network, the convolution layers, and the symmetry module. The recognition model based on a multi-view convolutional neural network is exhibited in Figure 7. Firstly, a transformation correction network is designed to correct the multi-view 2.5D point cloud data spatially. Then, two convolutional layers are employed to extract the features and a transformation correction network is designed to correct the extracted features spatially afterward. After that, a symmetric module composed of three convolution layers and a max pooling layer is applied to make the recognition results invariant to the permutation of the input. Afterward, a view fusion network is used to aggregate the features of all views into a single and global feature descriptor. Finally, three fully connected layers are used to classify the categories of objects.

The convolution kernel in a convolutional neural network reflects the local features of objects, and the degree of localization depends on the size of the convolution kernel. Because of the interaction among points, it is especially crucial to select an appropriate convolution kernel in the model to capture the local features among nearby points. This paper selects a 1 × 3 convolution kernel when convolving with the raw point clouds to synthesize three coordinates and uses the 1 × 1 convolution kernel when extracting the features initially. Lastly, it selects the 5 × 1 convolution kernel in the structure of the symmetric module to extract the local features.

## 5. Results

In order to evaluate the proposed method, we perform controlled experiments on two publicly available databases introduced by Zhirong Wu et al. [18]. One is the ModelNet10 database with 10 categories, which contains 4266 training samples and 908 testing samples. The other is the ModelNet40 database with 40 categories, which contains 9843 training samples and 2468 testing samples. This paper generates the multi-view 2.5D point cloud data corresponding to the ModelNet10 database and ModelNet40 database respectively as the experimental data and then it normalizes the multi-view 2.5D point cloud data into a unit sphere. In addition, this paper augments the training data from two aspects. One is about jittering each point by a Gaussian noise with a 0 mean and a 0.01 standard deviation. The other is about rotating each point at a random angle in [0, 2π] along the *y*-axis. We use a workstation with Core i7 CPU and 16G RAM, which is equipped with NVIDIA TITAN XP GPU and an open source deep learning framework TensorFlow. Our model takes between five and six hours on ModelNet10 and between fourteen and fifteen hours on ModelNet40 to train on a 12 GB GPU. Besides, our model takes 0.005 s to test an input sample.

### 5.1. The Effect of the Number of Points on Recognition Results

**Figure 8:**
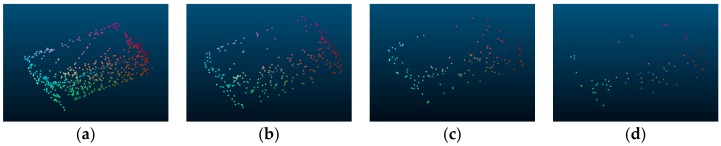
The 2.5D point clouds under various numbers of points from the same view. (**a**) the 2.5D point cloud with 512 points; (**b**) the 2.5D point cloud with 256 points; (**c**) the 2.5D point cloud with 128 points; (**d**) the 2.5D point cloud 64 points.

In order to analyze the effect of the number of points on recognition accuracy, we test the multi-view 2.5D point cloud data with 64, 128, 256, and 512 points, respectively. Figure 8 presents some 2.5D point clouds with different numbers of points.

The experimental results for different numbers of points on ModelNet10 are shown below.

**Figure 9:**
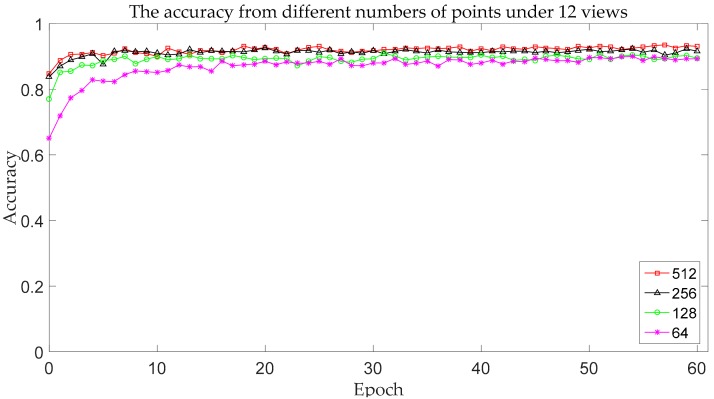
The accuracy change curve with different numbers of points.

It can be seen from Table 1 and Figure 9 that when there are 50%, 75%, and 87.5% points missing, the accuracy drops by 1.3%, 2.8%, and 3.6% respectively. This shows that when the number of points is enough to reflect the shape of the object, the resolution of the point clouds has little effect on recognition, which proves that the model proposed is suitable for both low-resolution and high-resolution point clouds.

### 5.2. The Effect of the Number of Views on Recognition Results

In order to analyze the effect of the number of views on recognition accuracy, we test the multi-view 2.5D point cloud data with 1 view, 6 views, and 12 views, respectively.

The experimental results for different numbers of views on ModelNet10 are shown below.

**Figure 10:**
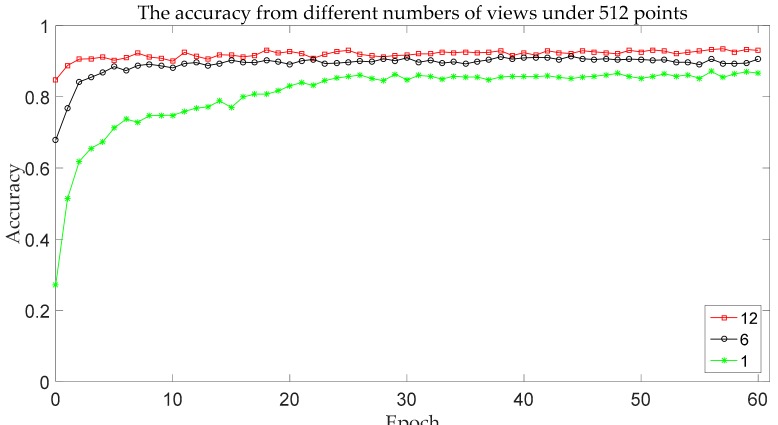
The accuracy change curve for different numbers of views.

As shown in Table 2 and Figure 10, when the number of views reduces by 50%, the accuracy drops by 3.0%. When there is only one view, the recognition accuracy is just 86.6%. It turns out that our model is sensitive to the amount of shape information. The model proposed can make full use of the information of each view since each view reflects its local information. When the number of views decreases, part of the information is lost, the data expression ability is weakened, and the recognition accuracy is consequently reduced.

### 5.3. The Effectiveness of Symmetry Module, Transformation Correction Network, and View Fusion Network

To demonstrate the effectiveness of the symmetry module, the transformation correction network, and the view fusion network, we conduct a series of contrast experiments on whether they exist or not. First, to prove the effect of the symmetric module, we replace it with a common convolution-pooling structure. The convolution-pooling structure contains three convolution layers with kernel sizes of 5 × 1, 5 × 1, and 6 × 1, respectively, as well as three max-pooling layers with kernel sizes of 2 × 1, 2 × 1 and 2 × 1 respectively. Second, we carry out two sets of experiments with and without the transformation correction network. Lastly, we remove the view fusion network in the model. Instead of using 2.5D point clouds from all views as an input sample, we take each view as an input sample by separating multiple views. The comparison results are shown in Table 3 where all of the above give a marked performance boost, especially the view fusion network.

### 5.4. Comparison with Other Methods

In Table 4, we compare our method with the current state-of-the-art methods on ModelNet10 and ModelNet40.

Table 4 shows that our method outperforms both the method that represents 3D models as voxel grids and the method that projects 3D models to 2D images. In addition to considering the disorder and transformation of point clouds, it is mainly due to the fact that this paper presents a view fusion network which can make full use of the information from multiple views. As can be seen in Table 4, the accuracy drops by 3.0% on ModelNet10 and 1.1% on ModelNet40 when the view fusion network is replaced by a view-pooling layer. It shows that this paper proposes a more efficient fusion method than the view-pooling layer proposed by Hang Su et al. [20]. The fusion method in this paper has a stronger non-linear expressive ability and can learn to map the features of multiple views into a global feature descriptor through more appropriate parameters instead of simply combining each view together. When the number of views changes from 12 to 20, it only yields a small improvement in the accuracy of recognition. However, it comes with a higher computational cost and training time. In short, the excessive increase in the number of views and points brings redundant information along with a large amount of calculation and processing time, so the users should set the number of views and points reasonably through balancing the need of accuracy and the need of efficiency.

## 6. Conclusions and Outlook

In this paper, a point cloud recognition model based on a multi-view convolutional neural network is proposed in the light of the characteristics of lidar point clouds. Our model displays a stronger robustness through solving the disorder of point clouds and the influence of affine transformation on recognition. In addition, the key to our model is the use of a view fusion network that is able to fully exploit the information of point clouds. The proposed method in this paper can directly process lidar point clouds and adaptively fuse point clouds from multiple views at the same time, thus avoiding three-dimensional reconstruction and other point cloud preprocessing. More notably, it is also effective in recognizing low-resolution point clouds. Therefore, this method is applicable to real-world lidar data and can be widely used in the fields of unmanned vehicles, drones, etc. In the future work, we will find a new method to solve the disorder of points in order to avoid the loss of information caused by the symmetric module. Moreover, we hope to obtain effective recognition results with as few views as possible by measuring the importance of each view and then selecting some of the most informative views, by which we will greatly reduce the processing time and achieve effective real-time 3D recognition and tracking.

## Figures and Tables

**Table 1:** The accuracy from different numbers of points under the 12 views.

The Number of Points	Accuracy
64	89.9%
128	90.7%
256	92.2%
512	93.5%

**Table 2:** The accuracy with different numbers of views under 512 points.

The Number of Views	Accuracy
1	86.6%
6	90.5%
12	93.5%

**Table 3:** The effects of symmetry function, transformation correction network, and view fusion network.

Method	Accuracy of ModelNet10	Accuracy of ModelNet40
all	93.5%	90.5%
no symmetry module	86.7%	76.1%
no TCN ^1^	88.9%	82.0%
no view fusion network	84.3%	73.3%

^1^ represents the transformation correction network.

**Table 4:** The classification accuracies for various methods on the ModelNet10 and ModelNet40 datasets.

Method	Views	ModelNet10	ModelNet40
SPH [29]	--	79.79%	68.23%
LFD [30]	--	79.87%	75.47%
3D ShapeNets [18]	--	83.54%	77.32%
DeepPano [31]	--	88.66%	82.54%
VoxNet [17]	--	92.0%	83.0%
PointNet [19]	--	--	89.2%
MVCNN [20]	12	--	89.9%
MVCNN [20]	80	--	90.1%
Our Methods ^1^	12	90.5%	89.4%
Our Methods ^2^	12	93.5%	90.5%
Our Methods ^2^	20	94.1%	90.8%

^1^ Using the view-pooling layer proposed in MVCNN. ^2^ Using the view fusion network proposed in this paper.
